# Glycine attenuates myocardial ischemia-reperfusion injury by inhibiting myocardial apoptosis in rats

**DOI:** 10.7555/JBR.26.20110124

**Published:** 2012-06-29

**Authors:** Xiaozheng Zhong, Xiaoyu Li, Lingling Qian, Yiming Xu, Yan Lu, Jing Zhang, Nan Li, Xudong Zhu, Jingjing Ben, Qing Yang, Qi Chen

**Affiliations:** a Atherosclerosis Research Center, Key Laboratory of Cardiovascular Disease and Molecular Intervention, Nanjing Medical University, Nanjing, Jiangsu 210029, China;; b State Key Laboratory of Reproductive Medicine, Nanjing Medical University, Nanjing, Jiangsu 210029, China.

**Keywords:** glycine, glycine receptor α2 subunit, ischemia reperfusion, apoptosis, cardiomyocytes

## Abstract

Glycine is a well-documented cytoprotective agent. However, whether it has a protective effect against myocardial ischemia-reperfusion injury *in vivo* is still unknown. By using an open-chest anesthetized rat model, we found that glycine reduced the infarct size by 21% in ischemia-reperfusion injury rats compared with that in the vehicle-treated MI/R rats. The left ventricular ejection fraction and fractional shortening were increased by 19.11% and 30.98%, respectively, in glycine-treated rats. The plasma creatine kinase levels in ischemia-reperfusion injury rats decreased following glycine treatment. Importantly, administration of glycine significantly inhibited apoptosis in post-ischemia-reperfusion myocardium, which was accompanied by suppression of phosphorylated p38 mitogen-activated protein kinase and c-Jun NH_2_-terminal kinase, as well as the Fas ligand. These results suggest that glycine attenuates myocardial ischemia-reperfusion injury *in vivo* by inhibiting cardiomyocytes apoptosis.

## INTRODUCTION

Myocardial infarction (MI) is an important contributor to cardiovascular mortality. Early and sustained restoration of blood flow through the occluded coronary artery is currently the most effective therapy to limit infarct size and preserve cardiac function after acute MI. Nevertheless, reperfusion is considered as a double-edged sword as it can lead to worsening of tissue injury[1]. Ischemia-reperfusion (I/R) injury includes distinct phases of cellular injury with adenosine triphosphate (ATP) depletion, lactate accumulation and acidosis observed during ischemia, and the production of reactive oxygen and nitrogen species during reperfusion[2]. Myocardial protection against I/R injury becomes a primary goal of therapeutic intervention.

Glycine, a non-essential amino acid, protects mammalian cells against ischemic cell injury by preventing cellular membrane leakage. Glycine offers protection against I/R injury *in vivo* in the kidney[3], liver[4]–[6], and intestine[7]–[10]. The proposed protective mechanism of glycine against I/R injury in liver tissues involves chloride influx and subsequent membrane hyperpolarization by activation of glycine receptors in Kupffer cells, which reduces inflammatory responses and maintains cellular energy production[5]. Study regarding the effect of glycine on myocardial I/R injury is ongoing. Ruiz-Meana *et al*.[11] showed that glycine increased viability of the isolated cardiomyocytes and the isolated rat hearts after I/R injury by preventing mitochondrial swelling and calcein release. Right ventricular compliance after transplantation is significantly improved following glycine application[12]. The protective effect of glycine on the isolated rat heart is linked to an attenuation of calcium influx in a lipopolysaccharide (LPS)-induced cardiac function loss model[13]. However, whether glycine exerts a beneficial effect on myocardial injury after I/R *in vivo* is unknown.

We investigated the effect of glycine on rat myocardial I/R injury *in vivo* in this study. The morphological and functional changes in the myocardium, as well as changes in relevant signaling pathways, were evaluated to gain a better insight into the specific mechanisms. We demonstrated for the first time that glycine could attenuate I/R-induced myocardial injury by inhibiting apoptosis in rats.

## MATERIALS AND METHODS

### Materials

Glycine, 2, 3, 5-triphenyltetrazolium chloride (TTC), Evans blue solution, mouse anti-β-actin, and rabbit anti-FITC antibody were purchased from Sigma (St. Louis, MO, USA). Chloral hydrate, atropine, and saline were supplied by Nanjing Chemical Reagent Co., Ltd. (Nanjing, China). The BCA protein assay kit was obtained from Pierce (Rockford, IL, USA). Terminal deoxynucleotidyl transferase dUTP nick end labeling (TUNEL) kit was purchased from Promega (Madison, WI, USA). Enhanced chemiluminescence (ECL) reagent was obtained from Amersham Biosciences (Piscataway, NJ, USA). The creatine kinase (CK) detection kits were purchased from Nanjing Jiancheng Bioengineering Institute (Nanjing, China). Rabbit antibodies against p38, mitogen-activated protein kinase (MAPK), or c-Jun NH_2_-terminal kinase (JNK) were obtained from Cell Signaling Technology (Beverly, MA, USA). Rabbit anti-glycine receptor (GlyR) α1/2 antibody was obtained from Abcam (Cambridge, UK).

### Animal treatment

Male Sprague-Dawley rats, each weighing 250±30 g, were purchased from the Animal Center of Nanjing Medical University. All surgical procedures were carried out according to the Guide for the Care and Use of Laboratory Animals, National Institute of Health, USA and all procedures were approved by the Institutional Animal Care and Use Committee of Nanjing Medical University. Rats were anesthetized with 10% chloral hydrate (0.35 mL/100 g body weight) and ventilated by using a positive-pressure respirator for small animals. After left thoracotomy, the left anterior descending coronary artery was occluded with a 6-0 silk suture. The body temperature of rats was measured and maintained at 37°C by placing the rats on a heating pad. After occlusion for 30 min, the suture was loosened and the myocardium was reperfused. Sham-operated rats underwent identical surgery but the suture was not tightened around the coronary artery. Rats were administered intraperitoneally with glycine (0.5 mg/g body weight) or saline 1 h before the operation.

### Quantification of myocardial infarct size

After 6 h of reperfusion, the coronary artery was again occluded. To map the risk areas for ischemia, 1% Evans blue solution was infused through the left jugular vein. Each heart was cut horizontally to yield 4 or 5 serial cross sections, of approximately 0.2 cm thick each section. The infarct areas were detected by incubating the sections with a 1.5% TTC solution for 10 min at 37°C. The infarct size was presented as the left ventricular infarct area in the ischemic area at risk.

### Measurement of myocardial function

Transthoracic echocardiography of the left ventricle was performed using echocardiography (GE Vivid 7) equipped with a 14-MHz phase array linear transducer, S12, allowing a 150 maximal sweep rate. All measurements were made by a single observer who was blinded to the identity of the tracings. All data were collected from 10 cardiac cycles. After rats were reperfused for 6 h, blood was collected and serum creatine kinase activity was determined using a kit according to the manufacturer's instructions (Nanjing Jiancheng Bioengineering Institute, Nanjing, China).

### Assessment of myocardial apoptosis and apoptotic signaling

Myocardial apoptosis was analyzed by TUNEL assay using an *in situ* cell death detection kit (Promega) according to the manufacturer's instructions. The index of apoptosis was expressed by the number of apoptotic cardiomyocytes/total number of cardiomyocytes counted×100%. Caspase-3 cleavage was assessed by Western blotting with an antibody against cleaved caspase-3 (Calbiochem, CA, USA).

### Isolation and primary culture of neonatal rat ventricular cardiomyocytes

Primary cultures of cardiomyocytes were prepared from the ventricles of 1- to 2-d-old Sprague-Dawley rats by enzymatic dissociation in 0.03% trypsin, 0.03% collagenase, and 20 µg/mL DNase I (Sigma). The cardiomyocytes were collected by differential adhesiveness. Cells were cultured in DMEM/F12 supplemented with 10% fetal bovine serum and penicillin (50 U/mL)/streptomycin (50 µg/mL) at 37°C in humid air with 5% CO_2_ .

Cardiomyocytes were grown on coverslips for 18-24 h at 37°C. After being fixed with 3% paraformaldehyde in PBS for 30 min at room temperature, cells were permeabilized with 0.1% Nonidet P-40 in PBS for 5 min, and blocked with 5% bovine serum albumin (BSA), 0.01% Tween 20/PBS (PBST-BSA) for 30 min. The primary antibody against GlyR α1/2 was incubated in PBST-BSA with cells overnight at 4°C, and then washed three times in PBST for 10-15 min each. After addition of the fluorochrome-conjugated secondary antibody (FITC-labeled goat anti-rabbit IgG) in PBST-BSA for 60 min, cells were washed three times in PBST for 10-15 min each. For controls, the primary antibody was omitted but the secondary antibody was added. Morphological observation was performed with a confocal microscope (Olympus, Japan). Pictures were obtained using sequential scanning, and the exposure settings and gain of laser were kept the same for each condition.

### Western blotting analysis

Proteins were extracted from cardiomyocytes or the area at risk of the heart. About 50 µg of the total protein were loaded per lane. The immunoblots were probed with antibodies against GlyR α1/2, p38 MAPK, JNK, or caspase-3 overnight at 4°C followed by incubation with the corresponding secondary antibodies at room temperature for 1 h.

### RNA isolation and gene expression analysis

Total RNA was extracted from hearts (for GlyR) or area at risk of the reperfused hearts (for Fas ligand and β-actin) using the Trizol reagent. One microgram total RNA was used for reverse transcription (RT). RNA samples were reverse-transcribed into cDNA using a Primescript TM RT reagent Kit. RT-PCR analysis was then performed by using the 7500 Real time PCR system (Applied Biosystems, CA, USA) with each amplified primer set under formulated conditions as described previously[14]. Deionized water was used to replace DNA template as negative controls. The primer sequences are available on request. Amplification data were analyzed and displayed by the 7500 Real time PCR system software. mRNA expression of *Fas ligand* was then quantitated in comparison with *β-actin* as their relative expression levels.

### Statistical analysis

All statistical analyses were conducted using GraphPad Prism Software version 5.0. All values were presented as mean±standard error of mean (SEM) or ±standard deviation (SD). Differences were tested for statistical significance using 2-tailed Student's *t* test or one-way analysis of variance (ANOVA) with Newman-Keuls test to determine the post-hoc differences. A *P*-value < 0.05 was considered statistically significant.

## RESULTS

### Glycine ameliorates MI after I/R injury

**Fig. 1:**
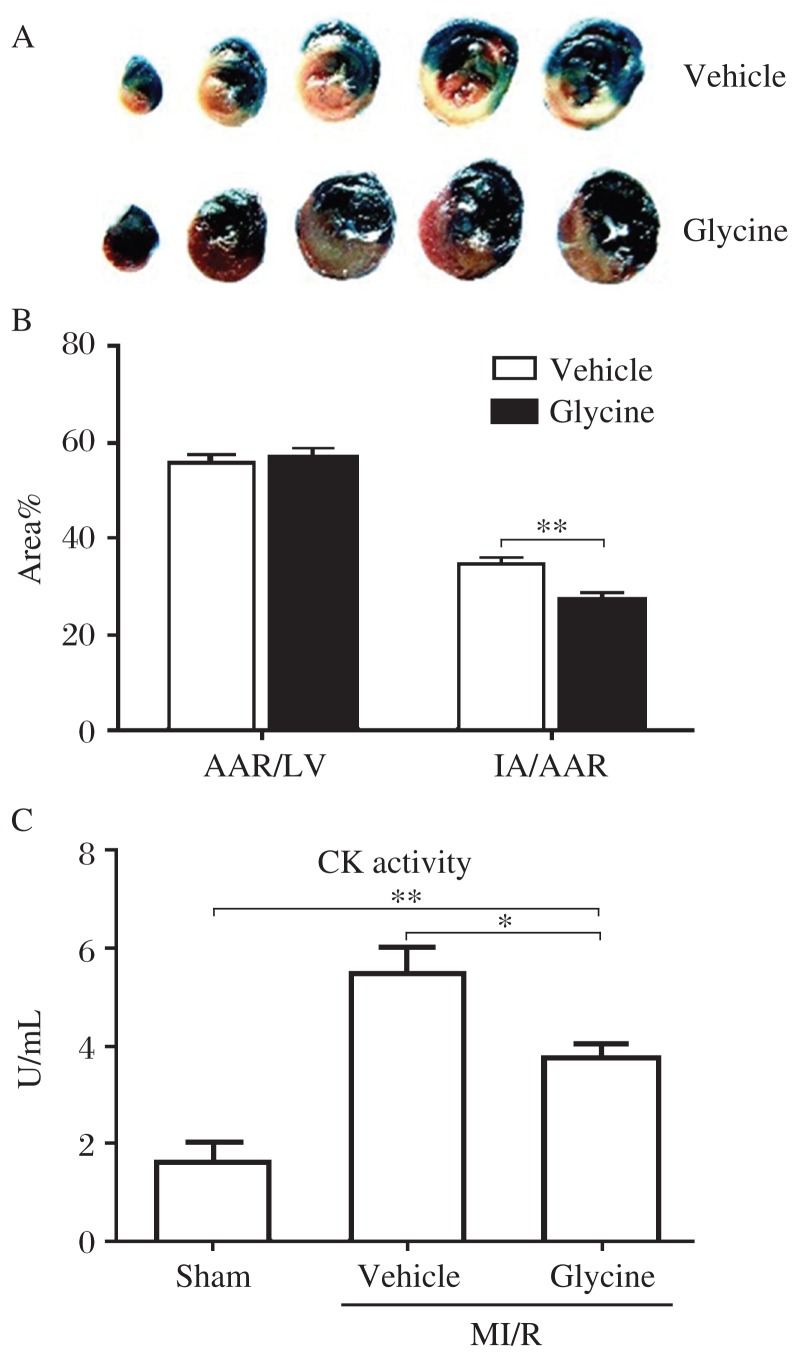
Effect of glycine on myocardial I/R injury in rats. A: Representative hearts stained with Evans blue/TCC (blue, nonischemic region; summation of red and white, ischemic region; white, infarct region) after 30 min of ischemia and 6 h of reperfusion. B: Quantification of infarct size after I/R. Data are expressed as mean±SEM, *n* = 6, **P < 0.01. C: Glycine treatment inhibits I/R-induced creatine kinase release in rats. Data are expressed as mean±SEM, *n* = 6, *P < 0.05, **P < 0.01. M: myocardial; I/R: ischemia-reperfusion; IA: infarct area; AAR: area at risk; LV: left ventricle.

To investigate the effect of glycine on myocardial I/R injury *in vivo*, we used Sprague-Dawley rats subjected to 30 min of myocardial ischemia followed by 6 h of reperfusion as an experimental model. The infarct size was visualized with Evans blue/TTC staining (***Fig. 1A***). When rats were pretreated with glycine, the ischemic area in the area at risk was significantly decreased by 21.74% (34.58%±1.22% in the vehicle group *vs* 27.06%±1.73% in the glycine treatment group, *n* = 6) after myocardial I/R. No significant difference in the area at risk in the left ventricle was found between these two groups (55.77%±1.37% *vs* 56.91%±1.71%) (***Fig. 1B***). Consistent with the alleviation of myocardial infarction, the increased plasma creatine kinase level, a biochemical marker of cell injury during reperfusion, was reduced by 31.42% in the glycine treatment group (3.75±0.28 U/mL) compared with that in rats receiving vehicle treatment (5.47±0.53 U/mL) after myocardial I/R (***Fig. 1C***). These results suggest that glycine ameliorates I/R injury in rat hearts.

### Glycine attenuates I/R-induced cardiac functions loss

**Fig. 2:**
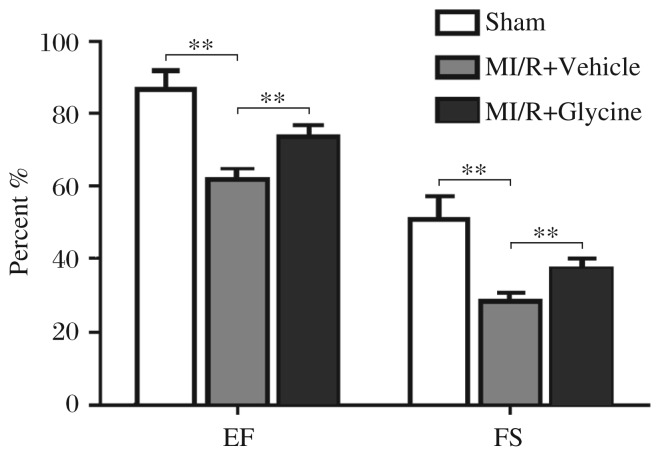
Glycine attenuates I/R-induced loss of cardiac functions. Rat ejection fraction and fractional shortening were measured by echocardiography. Data are expressed as mean±SEM, *n* = 6. **P < 0.01. M: myocardial; I/R: ischemia-reperfusion; EF: ejection fraction; FS: fractional shortening.

Myocardial I/R injury can cause loss of cardiac functions. We found that the left ventricular ejection fraction was dramatically repressed by 28.91% (86.80%±2.07% *vs* 61.67%±1.26%) and the left ventricular fractional shortening was reduced by 44.33% (51.20%±2.56% *vs* 28.50%±0.76%) in myocardial I/R rats compared with those in the sham group. Glycine treatment increased the left ventricular ejection fraction by 19.11% (from 61.67%±1.26% to 73.49%±1.39%) and the left ventricular fractional shortening by 30.98% (from 28.50%±0.76% to 37.33%±1.11%) in myocardial I/R rats (***Fig. 2***). No significant difference in cardiac diastolic parameters like the left ventricular end-diastolic volume (0.34±0.10 *vs* 0.30±0.11 ml), interventricular septal depth (1.39±0.19 *vs* 1.60±0.39 cm), or left ventricular end-diastole posterior wall thickness (1.40±0.16 *vs* 1.71±0.37 cm) was detected between the glycine- and vehicle-treated rats. Therefore, treatment with glycine may improve cardiac contractile performance of rats after I/R.

### Glycine blunts I/R-induced GlyR α2 up-regulation in cardiomyocytes

**Fig. 3:**
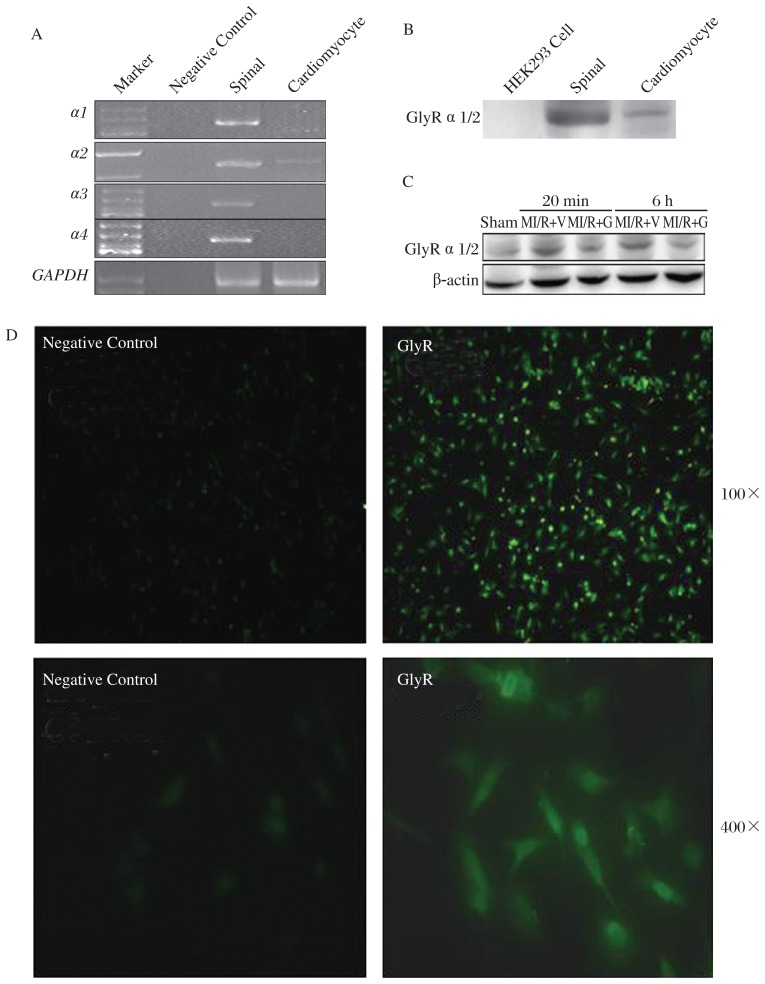
Glycine blunts I/R-induced GlyR α2 up-regulation in rat cardiomyocytes. A: mRNA expression of *GlyR* α subunits measured by RT-PCR. B: protein expression of GlyR α2 in cardiomyocytes measured by Western blotting. The cell lysate of HEK293 cells is used as a negative control. Proteins extracted from rat spinal cord are used as a positive control. C: Western blotting analysis of GlyR α2 in rat hearts. Rats are exposed to sham-operation or I/R surgery receiving vehicle or glycine treatment. Hearts are collected 20 min or 6 h after reperfusion and and proteins are extracted for Western blotting analysis. D: Immunofluorescence staining of GlyR α2 in primary rat cardiomyocytes. M: myocardial; I/R: ischemia-reperfusion; V: vehicle; G: glycine.

Glycine is a ligand of GlyR. To identify whether GlyR is requisite for protection of glycine against myocardial I/R injury, we firstly examined GlyR expression in cardiomyocytes. As shown in ***Fig. 3A***, the mRNAs of *GlyR* *α1,* *α2,* *α3* and *α4* subunits were expressed in spinal tissues but only *GlyR* *α2* mRNA was detected in rat myocardial tissues measured by a semi-quantitative RT-PCR. Western blotting analysis (***Fig. 3B***) and immunofluorescence staining (***Fig. 3D***) confirmed the protein expression of GlyR α2 in primarily cultured neonatal rat cardiomyocytes.

Subsequently, we investigated the role of GlyR α2 in glycine protection against myocardial I/R injury in rats. Western blotting analysis revealed that the expression of GlyR α2 in the area at risk was up-regulated in rats 20 min and 6 h following I/R. However, the I/R-induced up-regulation of GlyR α2 in cardiomyocytes was remarkably repressed following glycine treatment (***Fig. 3C***), indicating that GlyR α2 in hearts may be involved in the beneficial effect of glycine on I/R injury.

### Glycine inhibits I/R-induced myocardial apoptosis

**Fig. 4:**
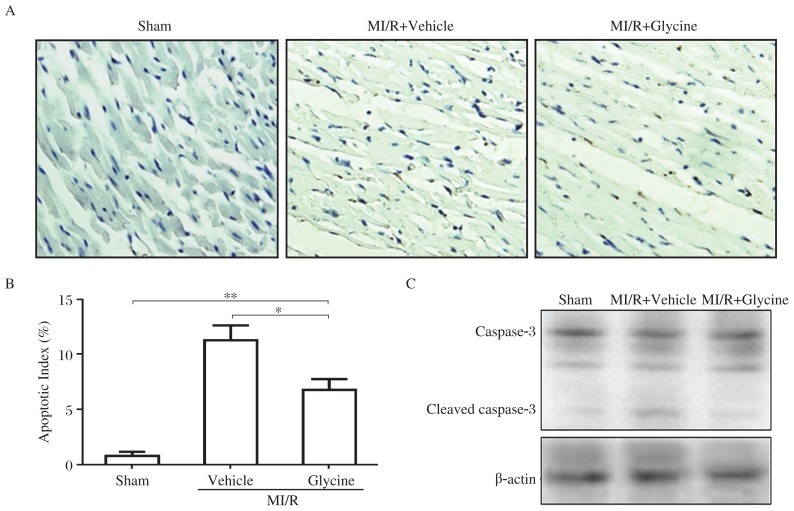
Glycine inhibits I/R-induced apoptosis in hearts. A: Representative TUNEL staining of rat heart. All myocardial tissues are isolated from the area at risk in rats and embedded in paraffin. B: Apoptotic rat myocardiocytes. Cells are photographed and evaluated under a microscope at a magnification of 200× in 10 different fields, of at least 1,000 cells in each field. Index of apoptosis is expressed as the percentage of TUNEL-positive cells relative to the total number of myocytes counted. Data are expressed as mean±SEM, *n* = 6, *P < 0.05, **P < 0.01. C: Western blotting analysis of caspase-3 in myocardial tissues. Proteins are extracted from the area at risk in heart. Representative blots from 3 separate experiments. M: myocardial; I/R: ischemia-reperfusion.

Our previous studies demonstrated that interaction between glycine and GlyR suppressed apoptosis-related signaling in Madin-Darby canine kidney (MDCK) cells[14]. To determine whether it constitutes the mechanism underlying glycine protection in heart, we assessed apoptosis in I/R myocardium. TUNEL staining showed that apoptotic cells were increased in the hearts of I/R rats. Treatment with glycine significantly reduced the number of TUNEL-positive cells compared with that in the vehicle treatment group after I/R injury (***Figs. 4A*** and ***4B***). Changes in caspase-3 cleavage in the area at risk measured by Western blotting were consistent with those of the apoptotic cells that up-regulated caspase-3 cleavage by I/R, which was decreased by glycine treatment (***Fig. 4C***).

**Fig. 5:**
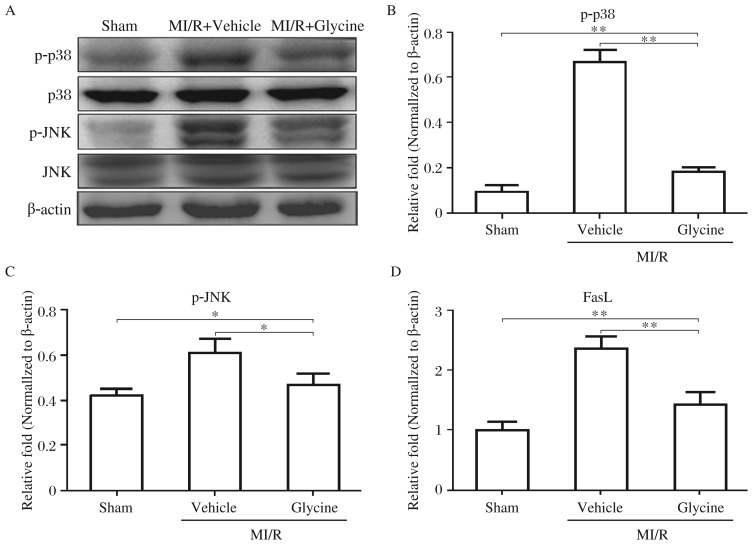
Effects of glycine on the signal molecules phosphorylation in I/R hearts. Hearts are harvested from the sham-operated rats or the rats subjected to 30 min of ischemia followed by 20 min of reperfusion receiving vehicle or glycine treatment. Proteins are extracted from the area at risk of hearts. Three independent experiments are performed. A, Representative Western blotting of phosphorylated p38 MAPK and JNK. Densitometric analysis of p-p38 MAPK (B) and p-JNK (C) bands normalized against β-actin; D: FasL ligand mRNA expression in hearts after MI/R. Hearts tissues are harvested after 30 min of ischemia followed by 20 min of reperfusion. Total RNA preparation is immediately made. *Fas ligand* mRNA analysis is made by quantitative RT-PCR. Data are expressed as mean±SD, *n* = 6, *P < 0.05, **P < 0.01. M: myocardial; I/R: ischemia-reperfusion.

In addition, we further probed the underlying signaling pathways. As shown in ***Figs. 5A*** to ***5C***, p38 MAPK and JNK were significantly activated after myocardium I/R. Glycine inhibited the activation of these two molecules. Furthermore, the results showed that the up-regulated *Fas ligand* mRNA expression in I/R myocardium was dramatically diminished by glycine treatment (***Fig. 5D***). These results suggest that inhibitory effect of glycine on cardiomyocyte apoptosis after I/R may be mediated by depression of p38 MAPK, JNK, and Fas ligand signaling pathways.

## DISCUSSION

Glycine is an essentially non-toxic physiological compound. Use of glycine at a single dose of 0.8 g/kg body weight per day is tolerated without any serious adverse effects in healthy human controls[15],[16]. Glycine is also used as a co-agonist for *N*-methyl-D-aspartate (NMDA) receptor in rats by intraperitoneal injection at a dose of 0.8 g/kg body weight[17]. The half-life of glycine after intravenous infusion or body cavity irrigation depends on the dose administered, which varies from 0.5-4 h[18]. Based on these reports, we chose the treatment protocol of intraperitoneal administration of glycine at a dose of 0.5 mg/g body weight with 1 h before the operation. It should be safe to rats, although lower doses of glycine (5, 10, 20, or 75 mg/kg boy weight) infused intravenously can be used to antagonize small intestine I/R injury[10].

The present study demonstrated that treatment with a single dose of glycine significantly reduced the infarct size induced by I/R in rat hearts. We demonstrated that the cardioprotective features of glycine, a known inhibitory neurotransmitter in the central nervous system, might be mediated by an anti-apoptotic mechanism in the ischemic myocardium. The anti-apoptotic mechanism has been found to work in antagonistic effects of glycine against rat mesenteric[8] and liver I/R injury[19]. However, whether glycine inhibits I/R-induced myocardiocyte apoptosis is not fully elucidated. A trial suggested that sublingual glycine treatment started within 6 h after the onset of acute ischemic stroke in the carotid artery territory could exert favorable clinical effects through attenuating neuron death[20]. But another study found that glycine did not add to the beneficial effect of preoperative oral immune-enhancing nutrition supplements in high-risk cardiac surgery patients[21]. Our findings showed clearly that the anti-apoptotic property of glycine might constitute a therapeutic mechanism of glycine to reduce I/R-induced MI, repress creatine kinase release from the heart, and improve cardiac functions.

Both the extrinsic and intrinsic apoptotic pathways are critical in the pathogenesis of MI. Our previous study indicated that glycine protected ATP-depleted MDCK cells against cell death by inhibiting p38 MAPK phosphorylation[14]. p38 MAPK and JNK/stress-activated protein kinase (SAPK) are strongly activated by stress and inflammatory cytokines, which are increased in I/R injury[22],[23]. The activated p38 MAPK and JNK/SAPK further phosphorylate transcription factors and cytoplasmic proteins, leading to the expression of adhesion molecules and cytokines, increased neutrophil activation, and expression of Fas ligand and other pro-apoptotic proteins[24]–[26]. Hence, myocardial apoptosis and necrosis occur[27]. Fas is a death receptor belonging to the tumor necrosis factor gene superfamily[28]. Fas-deficient mice exhibit marked reductions in infarct size following I/R compared with controls[29]. Fas receptor and Fas ligand are constitutively expressed in the myocardium[30]. Thus, glycine may attenuate I/R-induced myocardial injury by inhibiting cardiomyocyte apoptosis, in which activation of p38-MAPK and JNK and FasL expression are suppressed. Besides, other beneficial effects such as anti-inflammatory effect, immune regulation, and direct cell membrane protection may also be the protective mechanisms of glycine[31],[32].

It has been demonstrated that the cytoprotective effect of glycine is mediated *via* GlyR in MDCK cells[33]. GlyR is a pentameric protein belonging to the Cys-loop family of ligand-gated ion channels[34]. There are four different subtypes of α (α1-α4) and one β subunit, encoded by separate genes, in most species. GlyR α subunits are ligand-binding subunits, while β subunit is a structural subunit and gephyrin, a cytoplasmic anchoring protein[35]. The physiological role of GlyR α2 has not been extensively investigated. It is believed that GlyR α2 regulates interneuron differentiation during zebrafish spinal network development[36]. Our findings indicated that cardiomyocytes expressed GlyR α2 rather than GlyR α1, which was reported to exist in myocardial cells[13]. Indeed, the immunochemical results from both studies are consistent because the same antibody recognizing both GlyR α1 and GlyR α2 fragments was used by both groups. However, our PCR assay distinguished different α subunits of GlyR in rat cardiomyocytes and found that GlyR α2, instead of GlyR α1, was up-regulated in I/R hearts. It is possible that acute ischemia elicits the rapid release of various reactive substances to activate GlyR α2 and trigger deleterious processes in heart tissues. Glycine treatment could partially blunt this overexpression of GlyR α2 because of receptor desensitization accompanied by the diminution in MI. However, the direct consequence of the interaction between glycine and GlyR α2 in cardiomyocytes needs to be further explored.

In summary, it is proposed that glycine may act as an important cytoprotective agent in prevention of early myocardial I/R injury. This may ultimately provide a new therapy to reduce heart failure arising from MI.
